# *In Situ* Atomic-Scale Observation of Phase Evolution in Nickel Phosphide Nanoparticles

**DOI:** 10.1021/acs.nanolett.5c05549

**Published:** 2026-01-27

**Authors:** Kshipra Sharma, Tianyi Hu, Aryan Sankhla, Kimberly A. Dick

**Affiliations:** † Centre for Analysis and Synthesis, 5193 Lund University, 22100 Lund, Sweden; ‡ Wallenberg Initiative Materials Science for Sustainability, Centre for Analysis and Synthesis, Lund University, 22100 Lund, Sweden; § NanoLund, Lund University, 22100 Lund, Sweden; ∥ Institute for Frontier Materials on Earth and in Space, German Aerospace Center (DLR), D-51147 Cologne, Germany

**Keywords:** Nickel phosphide (Ni_*x*_P_*y*_) nanoparticles, Environmental transmission electron microscopy (ETEM), Phase evolution, Earth-abundant catalysts, Vapor−solid reaction

## Abstract

Nickel phosphides are promising earth-abundant, low-cost catalysts for hydrogen/oxygen evolution reactions and CO_2_ reduction. However, their formation mechanisms remain poorly understood and difficult to control. This particularly applies to mechanisms determining phase evolution, crystallinity, and morphology under reactive conditions, factors that critically influence catalytic activity and stability. Here, we employ environmental transmission electron microscopy to directly observe the conversion of nickel nanoparticles into nickel phosphide phases under controlled phosphine atmosphere and temperatures. A three-stage Ni-to-Ni_2_P conversion sequence is observed: (i) surface nucleation, (ii) rapid particle-size expansion, and (iii) crystallographic restructuring and faceting. Phase selectivity depends on the phosphine pressure and temperature: Ni_2_P forms at both low and high pressures, Ni_2_P and Ni_5_P_4_ coexist at intermediate pressure, and Ni_12_P_5_ emerges under no phosphine supply (residual phosphine may have remained) at elevated temperatures. We capture the temperature-driven Ni_2_P-to-Ni_12_P_5_ transition. These insights offer strategies to control the phase and morphology for improved catalytic performance.

## Graphical abstract

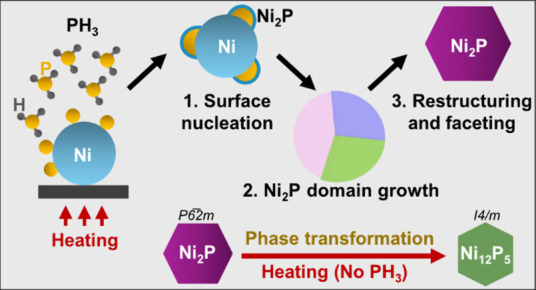

The development of efficient and robust catalysts remains a major challenge in advancing renewable energy technologies. Transition metal phosphides (TMPs), especially nickel phosphides (Ni_*x*_P_*y*_), have emerged as highly promising candidates for various catalytic processes, including hydrogen evolution reaction (HER), oxygen evolution reaction (OER), and CO_2_ reduction reaction (CO_2_RR), owing to their earth abundance, tunable electronic band structure, and high catalytic activity.^1–5^ A key advantage of Ni_*x*_P_*y*_ compounds lies in their rich phase diversity; this binary system hosts several thermodynamically stable phases, including Ni-rich compositions such as Ni_3_P, Ni_12_P_5_, Ni_2_P, and Ni_5_P_4_ as well as P-rich compositions such as NiP_3_, NiP_2_, etc.^1,6,7^ Each phase exhibits distinct crystal structures, chemical compositions, surface properties, and electronic configurations.^1,7^ These structural differences strongly influence their catalytic behavior, including binding strengths, final product selectivity, and reaction rates, providing an efficient way to tailor the catalytic properties of Ni_*x*_P_*y*_ for a variety of reactions.^1,6–9^ For example, Ni_2_P has shown promising HER performance, attributed to its optimal hydrogen adsorption energy, while Ni_5_P_4_ has been reported to outperform Ni_2_P in both acidic and alkaline media due to favorable surface energetics.^10–13^ Moreover, both Ni_2_P and Ni_12_P_5_ phases have demonstrated potential (as photo/electrocatalysts) for selective CO_2_RR toward multicarbon compounds.^4,5,13–16^ Beyond phase identity, factors such as morphology, crystallinity, and exposed facets of these phosphides also play a crucial role in defining their reactivity by influencing the distribution and accessibility of active sites.^10,11,17–19^

Despite their potential, a major challenge in Ni_*x*_P_*y*_ catalyst development is the ambiguity associated with synthesis parameters, which resulted in the lack of precise and independent control over the key structural characteristics in such catalysts. Moreover, the lack of clarity on how these characteristics interrelate limits the ability to use synthesis conditions as effective levers for achieving the desired phases and morphologies. Conventional synthesis methods, such as solid-state reactions, solution-phase synthesis, and chemical vapor deposition, often yield mixed-phase products.^20^ This presents two key challenges: first, catalytic performance may be compromised when specific phases or morphologies are relatively more active than the other, and second, it becomes difficult to isolate the contribution of individual phases or morphologies to the observed catalytic activity, especially when their formation cannot be precisely controlled in most studies. These conventional approaches provide limited insight into how synthesis variables (such as temperature, phosphine (PH_3_) pressure, etc.) govern formation mechanisms, morphological evolution, and phase stability. Most studies rely on postsynthesis characterization, which cannot directly capture the spatial–temporal evolution of formation processes. As a result, several overlapping and intermediate phenomena, such as nucleation, growth, structural rearrangement, morphological evolution, and phase transitions, remain largely unexplored and poorly controlled.

To address this gap, we present a direct, real-time investigation of nickel phosphide formation dynamics using an environmental transmission electron microscope (ETEM). By applying a solid–vapor phase reaction between Ni nanoparticles and PH_3_ gas under varying temperature and pressure conditions, we capture the nucleation, growth dynamics, crystallinity, and morphological evolution as well as phase transition processes of Ni_2_P at the atomic scale. This method enables us to directly track the real-time conversion of individual Ni nanoparticles into phase-pure or mixed-phase Ni-phosphides, including their crystallinity and morphological evolution. In this work, we aim to address two fundamental questions that are critical for understanding and achieving the controlled growth of Ni_*x*_P_*y*_ catalysts. First, how do synthesis parameters, particularly PH_3_ pressure and temperature, govern the selection and stability of different Ni_*x*_P_*y*_ phases as well as crystallinity and morphology during solid–vapor synthesis? Second, what are the real-time dynamics underlying the formation and transformation of these phases? By systematically investigating these aspects, we seek to establish a clearer relationship between synthesis conditions and structural outcomes, thereby enabling the rational design of phase-pure and structurally optimized Ni_*x*_P_*y*_ catalysts. Our real-time ETEM observations reveal the formation of three distinct stoichiometric phases, Ni_2_P, Ni_5_P_4_, and Ni_12_P_5_, depending on PH_3_ pressures at the sample and synthesis temperatures. The conversion of Ni nanoparticles into Ni_2_P follows a multistage pathway, initiating at the surface and progressing inward. This process involves (1) multiple Ni_2_P surface nucleation events on Ni, (2) rapid particle size expansion resembling popcorn-like growth, and (3) subsequent crystallographic restructuring and faceting. A further real-time transition from Ni_2_P to Ni_12_P_5_ at elevated temperatures was also been captured. These observations provide valuable insights into phase selection and transformation, formation dynamics, crystallinity, and morphological evolution in Ni-phosphides at the atomic scale, providing pathways for their controlled synthesis for targeted catalytic reactions.

To investigate the real-time formation dynamics of Ni_*x*_P_*y*_, we deposited Ni nanoparticles (average diameter of 30 nm) onto micro-electro-mechanical system (MEMS) heating chips using a custom-built spark discharge generator.^21^ The MEMS chips were then transferred individually to the ETEM, a dedicated TEM combined with a customized gas handling system,^22^ where they were exposed to PH_3_ at varying sample pressures (typical pressures for low: 1.31 Pa, medium: 7.15 Pa and, high: 26.2 Pa) at controlled synthesis temperatures (300 °C for low and 700 °C for high) for an optimized reaction time. The formation dynamics were captured by high-speed, high-resolution imaging during gas exposure. Crystal structure was identified by analyzing the acquired high-resolution images, while composition was monitored by energy-dispersive X-ray spectroscopy (EDX). EDX spectra were acquired at 300 °C with an acquisition time of 120 s for each spectrum. Additional experimental details, including sample preparation, electron microscopy imaging parameters, and PH_3_ flows in the reactor are provided in the Experimental Section of the Supporting Information (SI).

First, Ni nanoparticles were exposed to PH_3_ at a low pressure and a low synthesis temperature (300 °C). The particles transformed into phase-pure hexagonal Ni_2_P (space group: *P* 62 *m* ^23^), and the resulting Ni_2_P particle is shown in Figure 1a along the [412] zone axis. This phase remained stable, showing no structural changes even after prolonged exposure (∼60 min) under the same synthesis conditions. These observations imply that Ni_2_P is a stable and preferentially formed phase under low PH_3_ pressure and low temperature conditions, a trend consistent with the preferential formation of Ni_2_P observed as an initial phase under relatively mild reaction conditions in other synthesis methods.^24,25^

**Figure 1:**
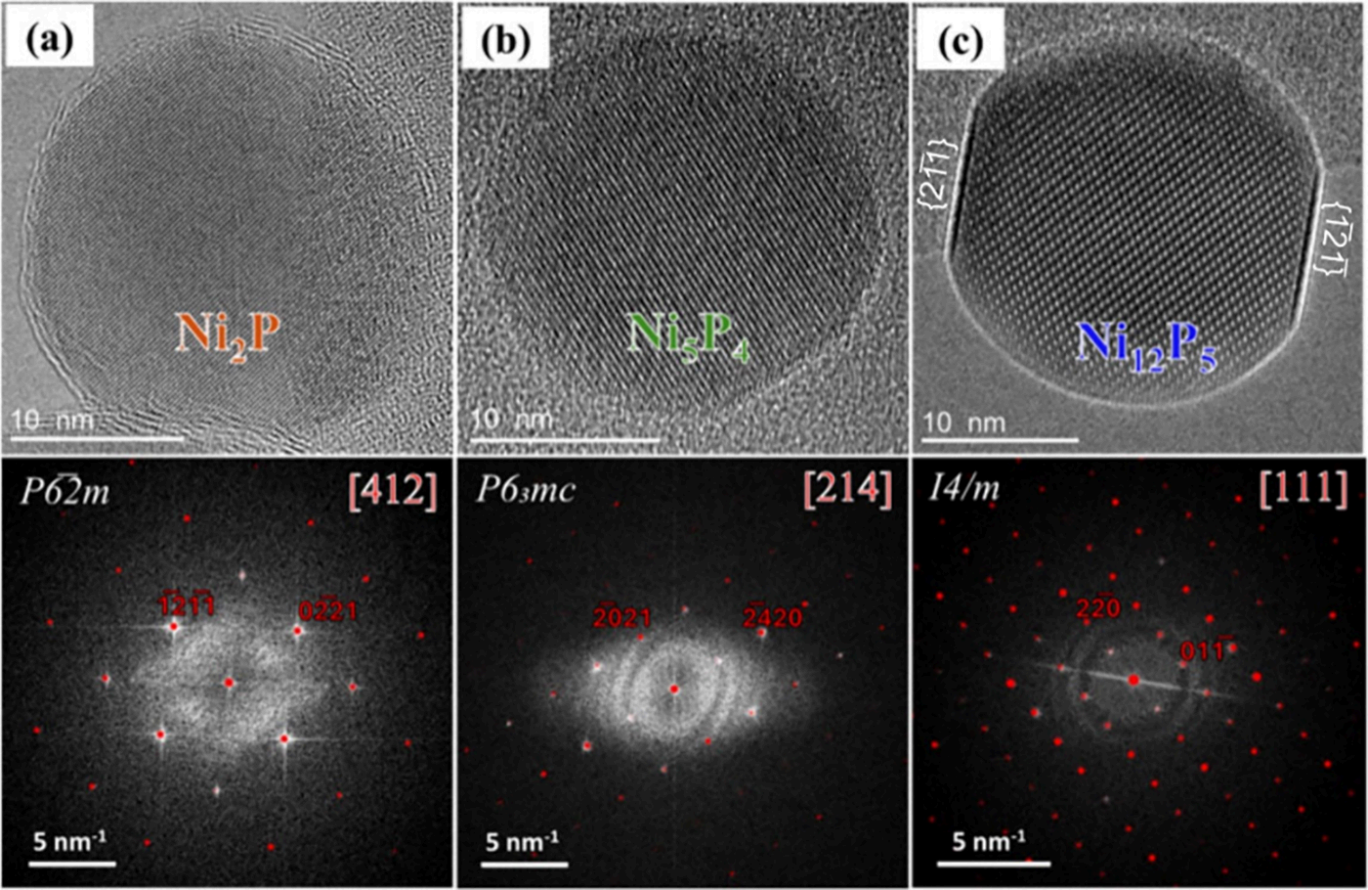
HRTEM images and corresponding power spectra of nickel phosphide nanoparticles formed by exposing Ni nanoparticles to different PH_3_ pressures and temperatures. The power spectra are compared with simulated diffraction patterns from crystallographic databases (ICSD), confirming the presence of three distinct phases: (a) Ni_2_P, (b) Ni_5_P_4_, and (c) Ni_12_P_5_. Images (a) and (b) were acquired at 300 °C with PH_3_ pressures of 1.31 and 7.15 Pa, respectively, while image (c) was acquired at 700 °C under no-PH_3_ supply. TEM imaging was performed with an electron dose rate of ∼3.4 × 10^3^ e^–^ Å^–2^ s^–1^ (dwell time: 0.5 s).

In contrast to low-pressure conditions, we then performed another experiment at medium pressure while maintaining the temperature at 300 °C. This resulted in the formation of two distinct phases, Ni_2_P and Ni_5_P_4_ (space group: *P6*_*3*_ *mc* ^26^). A representative Ni_5_P_4_ particle viewed along the [214] direction is shown in Figure 1b. The formation of both phases suggests that phase formation in Ni–P systems is highly sensitive to the chemical potential of phosphorus (through PH_3_). These observations support the idea that by tuning the thermodynamic conditions, such as PH_3_ pressure and temperature, the synthesis can be directed toward specific Ni_*x*_P_*y*_ phases. Notably, at this intermediate condition, we observe the coexistence of both Ni_2_P and Ni_5_P_4_ phases with an approximately equal distribution. Further, the temperature was increased from 300 to 700 °C in the absence of PH_3_ flow (PH_3_ supply to the chamber was stopped, although residual PH_3_ may have remained), and the particles transformed into a tetragonal Ni_12_P_5_ phase (space group: *I4/m* ^27^) (a representative Ni_12_P_5_ particle is shown in Figure 1c, oriented close to the [111] zone axis). This observation suggests that elevated temperatures can promote the transformation of Ni_2_P and/or Ni_5_P_4_ into a more Ni-rich Ni_12_P_5_ phase, likely driven by phosphorus loss [ref 28]. A detailed discussion of the thermal stability and phase transformation behavior of Ni_2_P under both PH_3_ and no-PH_3_ supply conditions is provided later in the study. Ni_12_P_5_ particles exhibited sharp-faceted morphologies (Figure 1c), possibly indicating surface energy minimization driven by thermodynamic effects at higher temperatures.

Lastly, exposing a fresh sample to both high PH_3_ pressure and a high temperature (700 °C) led to the re-emergence of the Ni_2_P phase. We hypothesize that Ni_2_P formation under both low and high PH_3_ pressure conditions may be governed by a critical phosphorus incorporation threshold, beyond which Ni_2_P becomes the thermodynamically favored phase. However, further investigation is needed to confirm this hypothesis.

EDX spectra of the nanoparticles exhibiting different phases are provided in Figure S1 (Supporting Information), confirming the elemental composition of the phases identified through crystallographic analysis. Table 1 summarizes the EDX-derived Ni:P atomic ratios. While the measured ratios are close to the expected stoichiometries of the assigned phases, small deviations are noticed. These deviations are often seen for nanoscale materials and can arise from low signal intensity, electron channeling, variations in particle thickness, defects, and other experimental factors.^29,30^ The measurements were acquired at 300 °C to capture the composition under the reaction conditions. While room-temperature EDX was not performed, the Ni–P phase diagram suggests phase stability upon cooling, though this needs to be confirmed for the nanoparticles. Phase identification in this study relies primarily on the match between experimental TEM power spectra (FFTs) and simulated electron diffraction patterns derived from the Inorganic Crystal Structure Database (ICSD) for the corresponding zone axes. Because the observed nickel phosphide phases, Ni_2_P, Ni_5_P_4_, and Ni_12_P_5_, exhibit distinct crystal structures but relatively close Ni:P ratios, high-resolution TEM and power spectra analysis provide a more reliable basis for phase identification, with EDX giving complementary confirmation of the overall Ni–P composition.

**Table 1:** Summary of EDX-Derived Ni:P Atomic Ratios for the Identified Phases^a^

Ni (at.%)	P (at.%)	Ni/P	Closet known phase	Figure ref.
65.5	34.5	∼1.9:1	Ni_2_P	S1(a)
63.4	36.6	∼1.7:1	Ni_5_P_4_	S1(b)
69.3	30.7	∼2.3:1	Ni_12_P_5_	S1(c)

a EDX spectra were acquired at 300 °C with an acquisition time of 120 s for each spectrum.

Further, to confirm that the observed phase evolution is representative, we performed HRTEM imaging, power spectra, and EDX analyses on approximately 25–30 nanoparticles from multiple, spatially separated regions of the MEMS chips. The data for three representative nanoparticles of each Ni_*x*_P_*y*_ phase are provided in the Supporting Information (Figures S2–S4). To evaluate the potential electron-beam effects, we analyzed nanoparticles located in previously unexposed regions of the MEMS chip. All particles exhibited the same phase evolution over the range of electron dose rates used (≈(1.7–3.4) × 10^3^ e^–^ Å^–2^ s^–1^), indicating that the observed transformations are primarily governed by PH_3_ pressures and temperatures rather than by electron beam irradiation.

After presenting an overview of the observed phases, we now return to examine in detail the dynamic process of Ni_2_P phase formation under low PH_3_ pressure and low temperature conditions. Figure 2 shows selected average frames from an HRTEM movie (Movie S1) that capture the real-time conversion of Ni nanoparticles into Ni_2_P inside the ETEM under these conditions. The frames in Figure 2 indicate a three-stage conversion sequence under consistent synthesis conditions: (I) localized nucleation of Ni_*x*_P_*y*_ at the surface of a Ni nanoparticle, (II) rapid growth and particle swelling, and (III) crystallinity and surface reconstruction with the emergence of facets.

**Figure 2:**
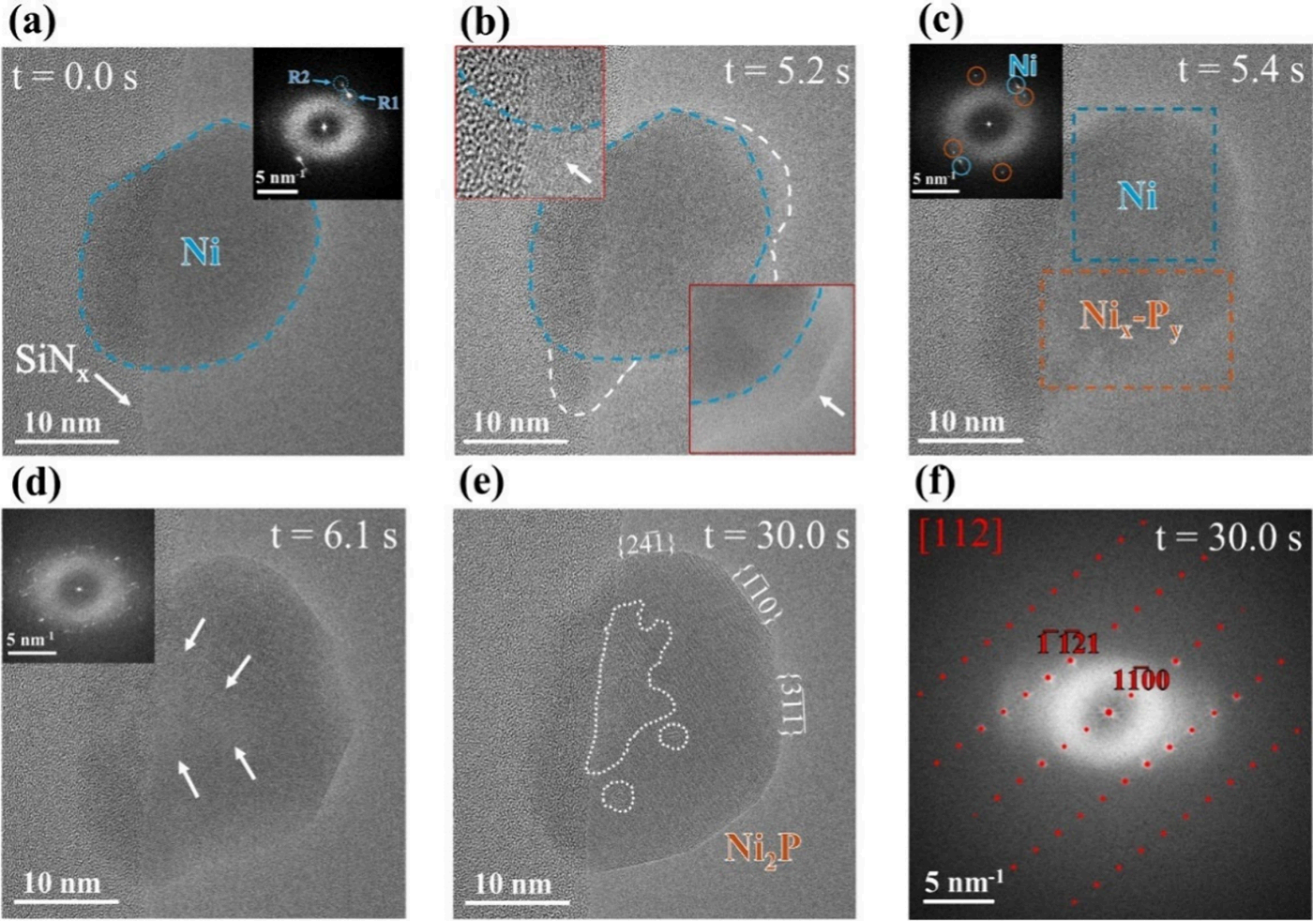
Selected HRTEM frames (each averaged over 4 frames with 4 skipped in between) from a time-resolved movie capturing the conversion of Ni nanoparticle into Ni_2_P under low PH_3_ pressure and low temperature conditions. (a) Ni nanoparticle before PH_3_ exposure. (b) Starting of localized nucleation at the particle surface. (c) Initiation of Ni_*x*_P_*y*_ domain growth from multiple nucleation sites. (d) Particle size expansion driven by the growth of crystalline Ni_*x*_P_*y*_ domains. (e) Complete conversion into single-phase Ni_2_P, followed by recrystallization into a single crystalline structure. (f) Power spectrum analysis confirms the Ni_2_P phase, matching well with a simulated diffraction pattern along the [112] zone axis. HRTEM movie recording conditions: *T* = 300 °C, PH_3_ partial pressure = 1.31 Pa, electron dose rate ≈ 3.4 × 10^3^ e^–^ Å^–2^ s^–1^ (dwell time: 0.5 s), frame rate = 25 frames/s.

In Figure 2a (*t* = 0.0 s), a Ni nanoparticle is shown at the edge of the silicon nitride (SiN_*x*_) membrane prior to PH_3_ exposure. The blue outline indicates the particle shape. A closer inspection of its power spectrum (inset of Figure 2a) points out small variations in the in-plane interplanar spacing, suggesting crystallographic heterogeneity within the particle. Spot masks were applied to two selected reflection sets (R1 and R2), and the corresponding inverse power spectra (Figure S5) show two regions with slightly different *d*-spacings (2.18 and 1.93 Å). Approximately 5 s after introducing PH_3_ at a low pressure at 300 °C, localized nucleation events began at the surface of nanoparticle (Figure 2b, *t* = 5.2 s), as marked with white outlines. Their localization at distinct sites suggests nonhomogeneous nucleation, likely corresponding to high-energy surface regions such as crystalline facets or defects. These nucleation events are likely preceded by PH_3_ adsorption and dissociation on the Ni surface, generating reactive phosphorus species that incorporate into near-surface layers. While these early stage processes are not directly resolved in ETEM, they align with reported reaction pathways involving PH_3_ decomposition and phosphorus incorporation into Ni, leading to Ni_*x*_P_*y*_ phase formation.^31^

Following nucleation, the growth of Ni_*x*_P_*y*_ domains initiated from the nucleation sites (just 0.2 s later, at *t* = 5.4 s), as shown in Figure 2c (highlighted by a square box in orange color). A set of additional reflection spots appeared in the power spectrum in addition to those corresponding to the initial Ni phase. The rest of the particle still displays characteristic Ni reflections (highlighted by a blue square). Spot masks were applied to isolate the Ni reflections and the newly emerging spots, and the corresponding inverse power spectrum images reveal the presence of small nickel phosphide domains growing from the nucleation sites (Figure S6). At *t* = 6.1 s, the entire particle underwent rapid radial expansion, and its diameter increased by approximately 8 nm (from ∼28 to ∼36 nm). This rapid transformation resembled a “popcorn-like” swelling behavior (Movie S1), suggesting an interdiffusion-driven phase formation process. We hypothesize that phosphorus atoms diffuse inward and nickel atoms diffuse outward, consistent with the Kirkendall effect. Such swelling behavior is also observed for interdiffusion-driven transformations in other nanoscale systems, where different atomic mobilities lead to void formation and particle expansion through the Kirkendall effect.^32–34^ While diffusion is strongly temperature-dependent, phosphorus diffusion in nickel is expected to be significantly faster than nickel self-diffusion at 300 °C, based on the reported trends in diffusion coefficients.^35^ This differential mobility can drive void formation and structural reorganization. At this stage, we observed lighter contrast regions (indicated by arrows in Figure 2d) within the particle, indicating nonuniform phosphorus penetration. This heterogeneity may result from confinement effects near the SiN_*x*_ membrane or progressive thinning of the Ni core due to outward Ni diffusion.

A detailed power spectrum analysis of the evolving particle at this stage is given in Figure 3(a,b), which revealed multiple Ni_*x*_P_*y*_ crystalline domains growing along different orientations. Four primary reflection sets (R1–R4) were identified, and their corresponding inverse power spectrum (Figure 3c–f) highlighted distinct crystal growth directions, which correlated with the initial nucleation sites identified earlier during the nucleation stage (Figure 2b). By *t* = ∼30 s (Figure 2e), the phase transformation was complete, and the pure Ni nanoparticle had fully converted into a Ni_*x*_P_*y*_. Power spectrum analysis of the particle matched well with a simulated electron diffraction pattern of Ni_2_P along the [112] zone axis, confirming the presence of a single crystalline phase (Figure 2f). The initial polycrystalline domains (Figure 2d and the detailed power spectrum analysis in Figure 3) had recombined, likely driven by the orientation of the dominant R1 nucleation domain. At the same time, the particle developed a well-defined faceted morphology with surface terminations such as {241}, {110}, and {311}. These facets are likely formed to reduce the surface energy, which is a common driving force during nanoparticle growth. While quantitative surface energy values for these high-index facets are not readily available, similar behavior has been reported in other Ni_2_P systems, where the facet stabilization is governed by local chemical potentials and kinetic growth conditions.^36^ Moreover, certain Ni_2_P facets, particularly (001) and (100), have been associated with enhanced catalytic performance.^37^ Although the facets we observed are different, and their impact on catalytic activity has not yet been identified, they may serve a similar purpose in enhancing catalytic efficiency. Further, to provide a semiquantitative indication of the transformation kinetics (from Ni-to-Ni_2_P), the projected particle area was tracked as a function of time from the ETEM movie (Movie S1), and the resulting area–time evolution is shown in the Supporting Information (Figure S7).

**Figure 3:**
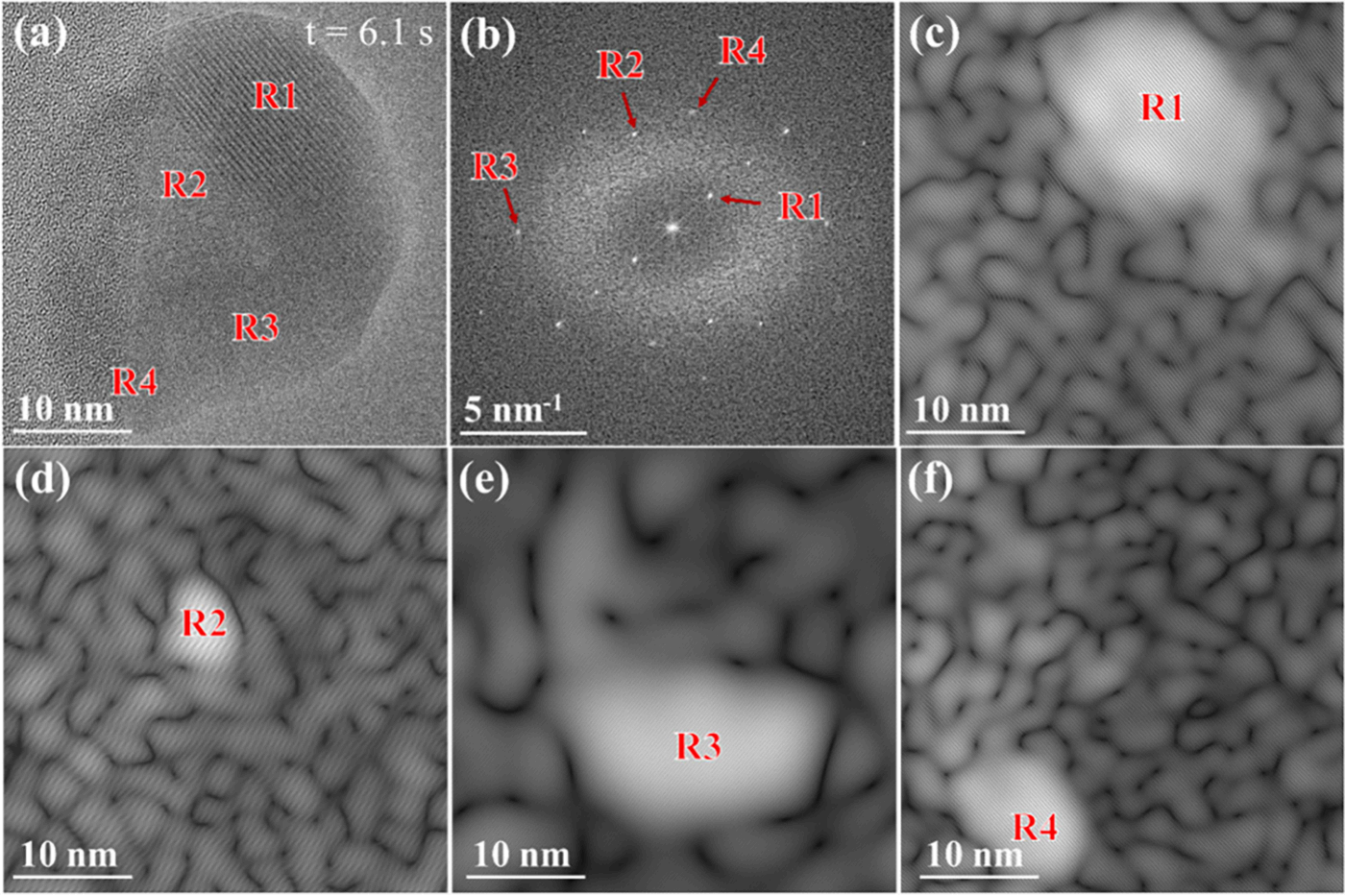
HRTEM image of the evolving nanoparticle during growth (as shown in Figure 2d), showing multiple Ni_*x*_P_*y*_ domains with varied orientations. (b) Corresponding power spectrum with distinct reflection sets (R1–R4). (c–f) Spot masks applied to individual reflection sets (R1–R4) and the resulting inverse power spectral analyses reveal distinct crystal growth directions. Frame acquisition conditions: *T* = 300 °C, PH_3_ pressure = 1.31 Pa, electron dose rate ≈ 3.4 × 10^3^ e^–^ Å^–2^ s^–1^ (dwell time: 0.5 s), frame rate = 25 frames/s.

At this stage, the particle appears fully crystallized, but the lighter contrast regions persisted, which possibly indicates localized voids from outward Ni diffusion through the Kirkendall effect or differences in thickness within the particle. Since crystallinity and morphology are known to influence catalytic behavior, the gradual evolution from polycrystalline to single-crystalline structure and the development of faceted morphology observed here may have important implications for catalyst performance. These findings suggest that crystallinity and surface morphology could potentially be tuned by controlling reaction time, temperature, and nucleation conditions during synthesis.

As introduced earlier (Figure 1c), the Ni_12_P_5_ phase evolved from the Ni_2_P and/or Ni_5_P_4_ phases at an elevated temperature under nominally PH_3_-free conditions (though residual PH_3_ might have been present in the chamber). We systematically investigated the thermal stability and phase transformation behavior of Ni_2_P nanoparticles across a temperature range of 300 to 700 °C under both low PH_3_ pressure and no-PH_3_ supply conditions. As shown in Figure 4a, under continuous low PH_3_, Ni_2_P remained structurally and compositionally stable throughout the temperature range. This stability was supported by consistent power spectra that matched the simulated diffraction patterns of Ni_2_P along the same viewing direction (Figure 4a, insets). However, a slight change in morphology was observed, as the particle facets became more pronounced (indicated by arrows) at higher temperatures, suggesting that facet structure can be influenced and potentially be tuned by thermal conditions.

**Figure 4:**
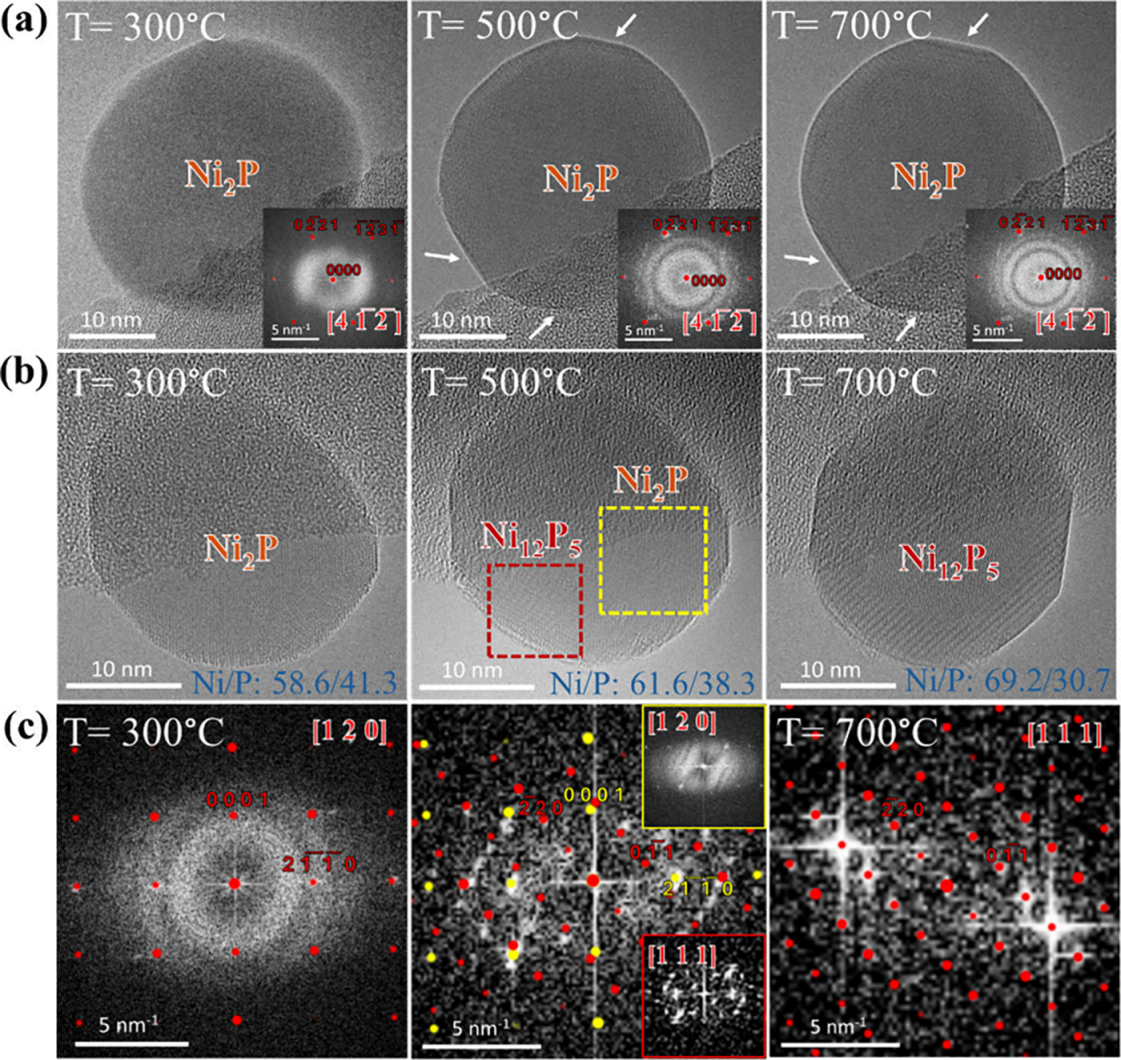
Temperature-dependent behavior of Ni_2_P under low PH_3_ pressure and no-PH_3_ supply conditions from 300 to 700 °C. (a) Ni_2_P remains structurally stable across the full temperature range under low PH_3_ pressure (1.31 Pa), with power spectra consistently matching those of simulated Ni_2_P. (b) Under no-PH_3_ supply condition, the phase remains stable up to 500 °C, where slight lattice changes (red square) suggest the beginning of phase transformation. (c) Power spectrum analysis at 500 °C revealing early Ni_12_P_5_ reflections (marked in red) from the highlighted region. By 700 °C, the transformation is complete and the particle fully converts to Ni_12_P_5_, confirmed by matching simulated and experimental power spectra. Images were acquired at an electron dose rate of 3.4 × 10^3^ e^–^ Å^–2^ s^–1^ (dwell time 0.5 s).

In contrast to the thermal behavior of Ni_2_P under low PH_3_ pressure, a remarkably different behavior was observed under no-PH_3_ supply condition (Figure 4b, different Ni_2_P particle). The Ni_2_P phase largely retained its structural and stoichiometric stability between 300 and 500 °C, as confirmed by power spectra matching the simulated Ni_2_P structure along the [120] zone axis (Figure 4c). However, upon heating to 700 °C, a significant transformation occurred structurally and morphologically. The Ni_2_P particle transformed into the Ni_12_P_5_ phase, confirmed by a clear match between the experimental and simulated power spectra of Ni_12_P_5_ (Figure 4c). This phase change is attributed to phosphorus loss from the Ni–P lattice at high temperatures in a phosphorus-deficient environment. Notably, slight changes at the particle surface were already detected at 500 °C (highlighted by a red square in Figure 4b). A detailed power spectrum analysis of the particle (Figure 4c) revealed the presence of Ni_12_P_5_ reflections (red spots), indicating the early stage of transformation from Ni_2_P to Ni_12_P_5_ at the particle surface at 500 °C. This transformation was completed by 700 °C with the particle fully converted into the Ni_12_P_5_ phase. The phase change is also supported by EDX analysis at these temperatures. The Ni/P ratios indicated on the images show a clear change, from values slightly shifted between Ni_2_P at 300 and 500 °C to a ratio approaching that of Ni_12_P_5_ at 700 °C. A similar trend has been observed in other synthesis methods like hydrothermal synthesis, where Ni_2_P initially forms as a kinetic product and gradually transforms into a more thermodynamically stable Ni_12_P_5_ phase in the presence of ligands over prolonged reaction times.^24,28^ However, our observations in a ligand-free vapor–solid reaction system demonstrate that elevated temperatures alone can facilitate the transformation in a short duration (∼30 min), pointing to a temperature-dependent phosphorus loss mechanism that may govern the phase transition.

In addition to the phase change, a clear morphological shift was observed. At 700 °C, the Ni_12_P_5_ particle developed a sharply faceted geometry, consistent with thermodynamically driven surface energy minimization at elevated temperatures, as seen earlier in this study. These findings support the hypothesis that the Ni_12_P_5_ phase observed earlier at an elevated temperature (Figure 1c) likely results from the thermal transformation of pre-existing Ni_2_P in a phosphorus-deficient environment. The emergence of well-defined facets also suggests that facet formation can be controllable, depending on the thermal environment and chemical potential. However, it remains unclear whether the coexisting Ni_5_P_4_ phase undergoes a similar transformation under these conditions. Further investigation is required to assess the thermal stability of Ni_5_P_4_ in a phosphorus-deficient atmosphere.

In summary, this *in situ* ETEM study offers key insights into the formation dynamics of nickel phosphide (Ni_*x*_P_*y*_) phases from Ni nanoparticles along with the morphological evolution under precisely controlled PH_3_ pressure and temperature conditions. We observed the formation of three distinct stoichiometric phases: Ni_2_P formed consistently under both low and high PH_3_ pressures, while Ni_5_P_4_ emerged along with Ni_2_P under intermediate PH_3_ pressure condition. Furthermore, we observed a three-stage Ni to Ni_2_P conversion sequence: (1) localized nucleation of Ni_2_P at the particle surface, (2) rapid particle swelling and interdiffusion between Ni and P, and (3) crystallographic and morphological restructuring with the emergence of facets. These observations highlight the critical roles of phosphorus diffusion and temperature in governing phase formation, crystallinity, and morphology, each of which is known to independently impact catalytic behavior. Understanding and controlling these parameters are therefore essential for optimizing the catalytic performance. Notably, the Ni_2_P phase remained structurally and compositionally stable up to 700 °C in the presence of a PH_3_ atmosphere. In contrast, under no-PH_3_ supply condition (residual phosphine may have remained in the chamber), Ni_2_P undergoes a progressive transformation at temperatures above 500 °C into the more Ni-rich Ni_12_P_5_ phase, likely driven by phosphorus loss at elevated temperatures. This transformation pathway can potentially be used to access distinct nickel phosphide phases through a controlled thermal treatment. By elucidating the temperature and phosphorus dependent mechanisms that govern phase, structural, and morphological transformations, this study establishes a pathway for the vapor–solid synthesis of nickel phosphide with different phases, tunable crystallinity, and morphology. Systematic correlation of reaction parameters, such as temperature and phosphorus pressure, with real-time structural evolution provides a practical way for controlling catalyst nanoparticle characteristics during synthesis. While the observations are related to the Ni_*x*_P_*y*_ system, the underlying methodology can be extended to other TMP nanoparticles formed via vapor–solid reactions. These insights are particularly promising for the advancement of Ni_*x*_P_*y*_ based catalysts in energy conversion and electrocatalysis applications.

## Supplementary Material





## Notes

Movie S1: Real-time conversion of Ni nanoparticle into Ni_2_P inside the ETEM (AVI)
Experimental details of Ni nanoparticle deposition; ETEM instrumentation and imaging conditions; HRTEM acquisition and analysis parameters; characterization and data analysis software; EDS measurement conditions and spectra; additional HRTEM images, power spectra, and inverse power spectra; supplementary experimental data (PDF)
