# Supplementary material for: In Situ Atomic-Scale Observation of Phase Evolution in Nickel Phosphide Nanoparticles
Source: Nano Lett. 2026 Jan 27;26(5):1774–81. doi: 10.1021/acs.nanolett.5c05549 (PMC12904070; doi:10.1021/acs.nanolett.5c05549)
Supplement: Supplementary file 2 [file nl5c05549_si_002.pdf]

Supplementary Information

In situ atomic-scale observation of phase evolution in nickel phosphide nanoparticles

Kshipra Sharma*[,1,2,3], Tianyi Hu[1,3], Aryan Sankhla[4], and Kimberly A. Dick*[,1,2,3]

[1]*Centre for Analysis and Synthesis, Lund University, 22100 Lund, Sweden*

[2]*Wallenberg Initiative Materials Science for Sustainability, Centre for Analysis and Synthesis, Lund University, 22100 Lund, Sweden*

[3]*NanoLund, Lund University, 22100 Lund, Sweden*

[4]*Institute for Frontier Materials on Earth and in Space, German Aerospace Center (DLR), D-51147 Cologne, Germany*

Experimental details

Ni nanoparticles deposition on MEMS-based heating chips

Nickel nanoparticles were generated using a custom-built spark discharge generator [23]. In this setup, nickel rods act as both anode and cathode to produce nanoparticles. The resulting particle agglomerates were transported by a H_2/N_2 carrier gas and subsequently passed through a furnace operated at 1100°C, leading to the formation of compact particles with different diameters. A differential mobility analyzer was used to select nanoparticles with a mobility diameter of approximately 30 nm. The size-selected nanoparticles were then electrostatically deposited onto micro-electro-mechanical system (MEMS)-based heating chips.

MEMS-based heating chips for *in situ* TEM experiments

The MEMS heating chips used for the *in situ* TEM experiments were supplied by Norcada. The chips allow temperatures of up to 1100°C with a homogeneous temperature profile in the central region of the membrane. Temperature control during the experiments was achieved using the Blaze software (Hitachi) operated in constant-resistance mode.

In situ TEM experiments: Imaging, movie and software used for characterization

ETEM experiments were performed using a Hitachi HF-3300S microscope operated at an acceleration voltage of 300 keV. The microscope is equipped with a cold field-emission gun and an image aberration corrector (CEOS BCOR). *In situ* ETEM movies and images were acquired using the integrated complementary metal-oxide-semiconductor (CMOS) camera (Gatan OneView IS), which enables high-speed recording of up to 300 frames/s. In this work, movies were recorded at 25 frames/s. The exported movie (Supplementary Movie S1) was processed using 20-frame averaging, 20-frame skipping, 4x time binning, and a playback rate of 5 frames/s to improve signal-to-noise ratio. The electron dose rate and dwell time used for imaging and movie acquisition are specified in the corresponding figure captions.

Elemental analysis was performed using EDX in TEM mode. EDX data were acquired at 300°C with an acquisition time of 120 s. Data was acquired and processed using AZtec from Oxford

Instruments Nanotechnology Tools Ltd. (Version 3.3). The background pressure near the sample was approximately 1.6 x 10^{-4} Pa under no-PH_3 supply condition. For the reaction, PH_3 gas was supplied through a gas-handling system equipped with mass flow controllers. This enables controlled supply of PH_3 directly to the heated region of the MEMS heating chip via a side-port injector integrated into the microscope column. The estimated pressure conditions at the sample and reactor used in the experiments are summarized in Table S1. All *in situ* experiments were conducted using a custom-built double-tilt MEMS holder (Hitachi). TEM images and movies were processed using Gatan Digital Micrograph and ImageJ. Phase identification was performed by comparing experimental power spectra derived from HRTEM images with simulated electron diffraction patterns obtained from the Inorganic Crystal Structure Database (ICSD) using Single Crystal software. Additional technical details of the ETEM setup at Lund University have been previously reported [24].

Set PH_3 flow (sccm) to ETEM	Estimated PH_3 pressure in reactor (Pa)	Estimated PH_3 pressure at sample (Pa)
0.2 (low)	0.22	1.31
2 (medium)	1.20	7.15
10 (high)	4.43	26.2

Table S1. PH_3 flow conditions used for the ETEM experiments.

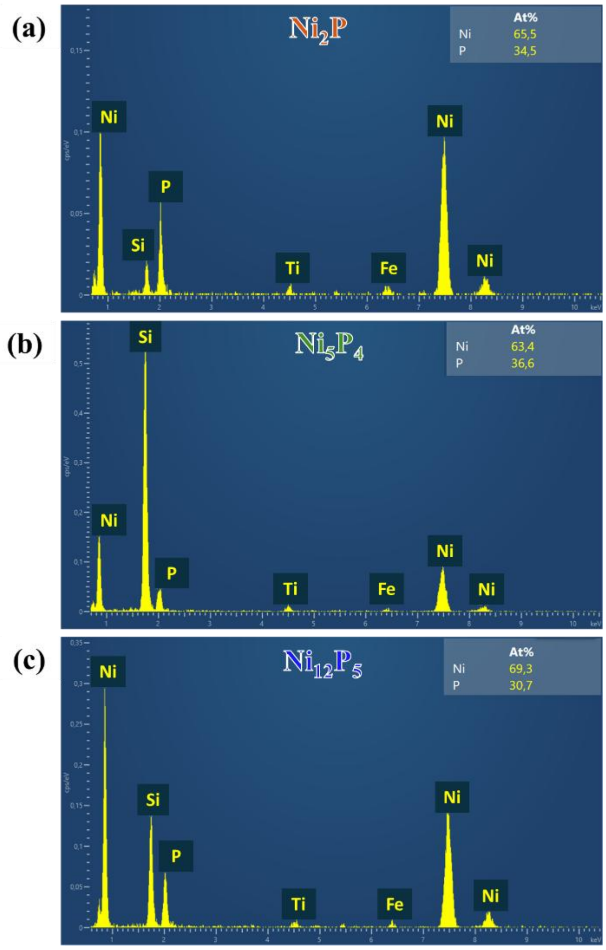

Figure S1. Energy-dispersive X-ray spectroscopy (EDX) spectra of three distinct nickel phosphide phases analyzed by AZtec software: (a) Ni_2P, (b) Ni_5P_4 and, (c) $Ni_{12}P_5$, formed by exposing Ni nanoparticles to varying PH_3 pressures and temperatures. Additional signals from Si, Ti, and Fe originate from the MEMS heating chip and sample holder. EDX data were acquired at 300 °C with an acquisition time of 120s for each spectrum.

(a)

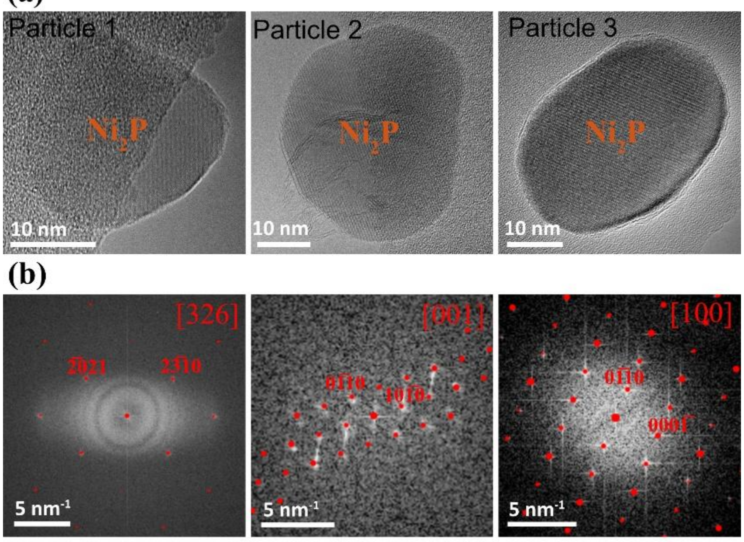

(c)

Element	Particle 1 (at%)	Particle 2 (at%)	Particle 3 (at%)	Std. dev. (at%)
Ni	64.1	63.4	62.6	0.75
P	35.8	36.6	37.4	0.80

Figure S2. Additional Ni_2P phase nanoparticles analyzed from different regions of the MEMS chip. (a) HRTEM images of Ni_2P nanoparticles. (b) Corresponding power spectra, compared with simulated electron diffraction patterns from the ICSD database, confirming the Ni_2P phase. (c) EDX-derived Ni:P atomic ratios for the particles, supporting the assigned Ni-P composition. All images were acquired at 300 °C under a low PH_3 pressure of 1.31 Pa, with an electron dose rate of approximately 1.7 x 10^3 e^- $Å^{-2}$ s^{-1} (dwell time: 1 s).

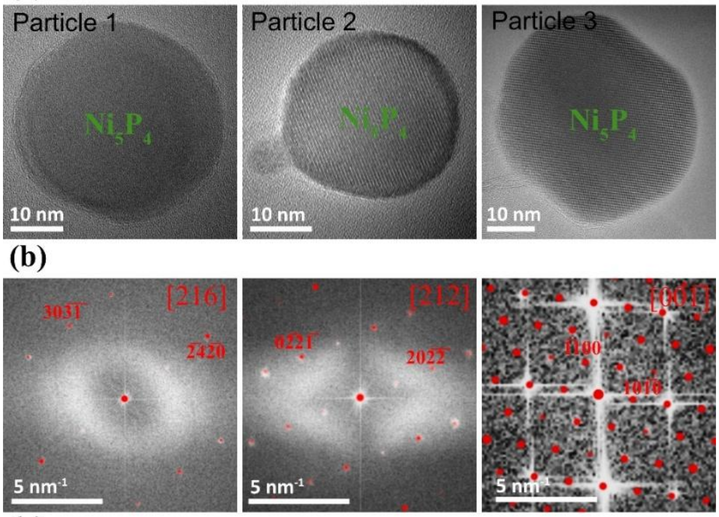

(c)

Element	Particle 1 (at%)	Particle 2 (at%)	Particle 3 (at%)	Std. dev. (at%)
Ni	54.2	54.6	55.1	0.48
P	45.7	45.4	44.8	0.48

Figure S3. Additional Ni_5P_4 phase nanoparticles analyzed from different regions of the MEMS chip. (a) HRTEM images of Ni_5P_4 nanoparticles. (b) Corresponding power spectra, compared with simulated electron diffraction patterns from the ICSD database, confirming the Ni_5P_4 phase. (c) EDX-derived Ni:P atomic ratios for the particles, supporting the assigned phase. All images were acquired at 300 °C under

a medium PH_3 partial pressure of 7.15 Pa, with an electron dose rate of approximately 1.7 x 10^3 e^- $Å^{-2}$ s^{-1} (dwell time: 1 s).

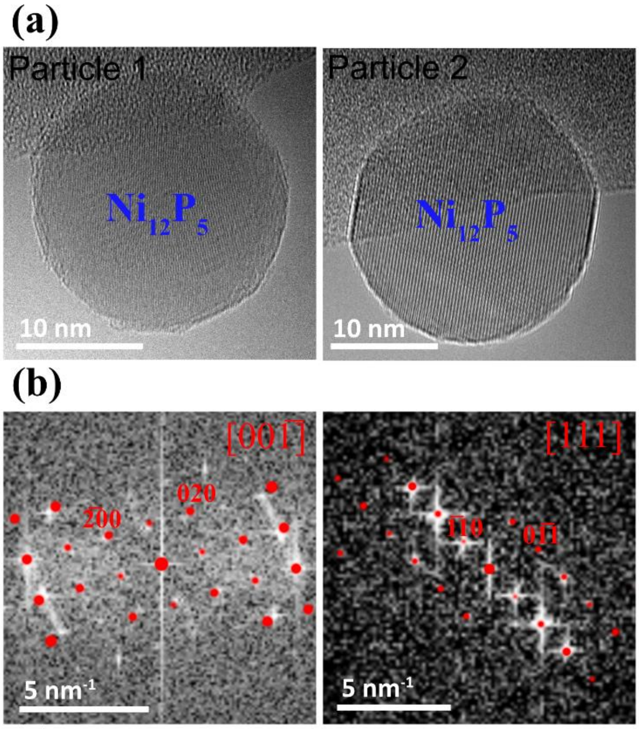

(c)

Element	Particle 1 (at%)	Particle 2 (at%)
Ni	69.6	71.7
P	30.4	28.3

Figure S4. Additional $Ni_{12}P_5$ phase nanoparticles analyzed from different regions of the MEMS chip. (a) HRTEM images of $Ni_{12}P_5$ nanoparticles. (b) Corresponding power spectra, compared with simulated electron diffraction patterns from the ICSD database, confirming the $Ni_{12}P_5$ phase. (c) EDX-derived Ni:P atomic ratios for the particles, supporting the assigned Ni-P composition. All images were acquired at 700 °C under no-PH_3 supply, with an electron dose rate of approximately 1.7 x 10^3 e^- $Å^{-2}$ s^{-1} (dwell time: 1 s).

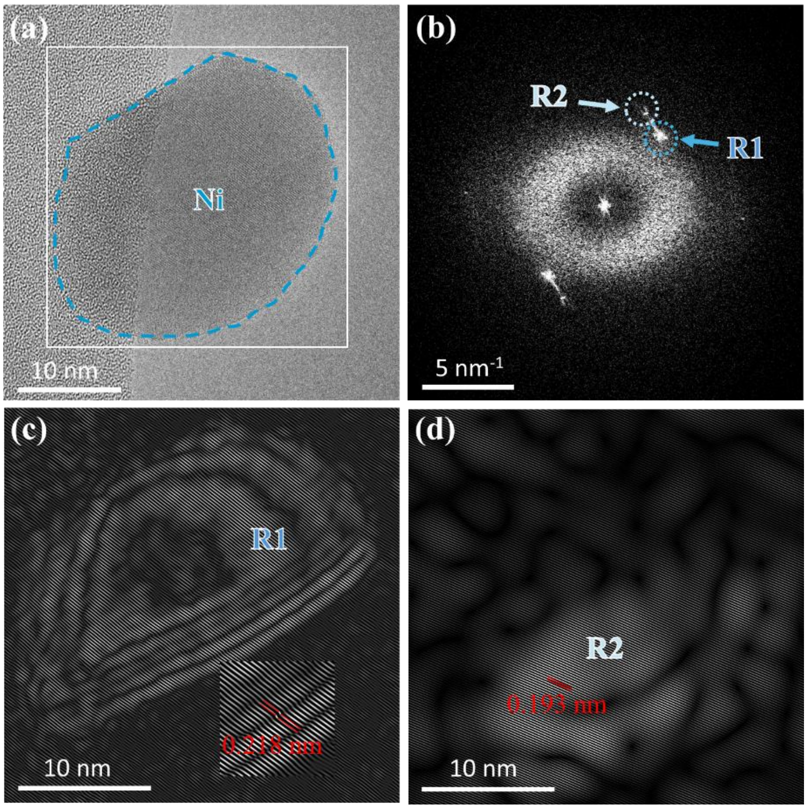

Figure S5. (a, b) TEM image of Ni nanoparticle prior to PH_3 exposure (shown in figure 2a) and its corresponding power spectrum, showing two distinct reflection sets (R1 and R2). (c, d) Inverse power spectra obtained using spot masks applied to R1 and R2, revealing slight variations in in-plane d-spacings (2.18 Å and 1.93 Å), indicating crystallographic heterogeneity. A dislocation is also visible in region R1. Imaging conditions: T = 300 °C, PH_3 partial pressure = 1.31 Pa, electron dose rate ≈ 3.4 x 10^3 e^- $Å^{-2}$ s^{-1} (dwell time: 0.5 s).

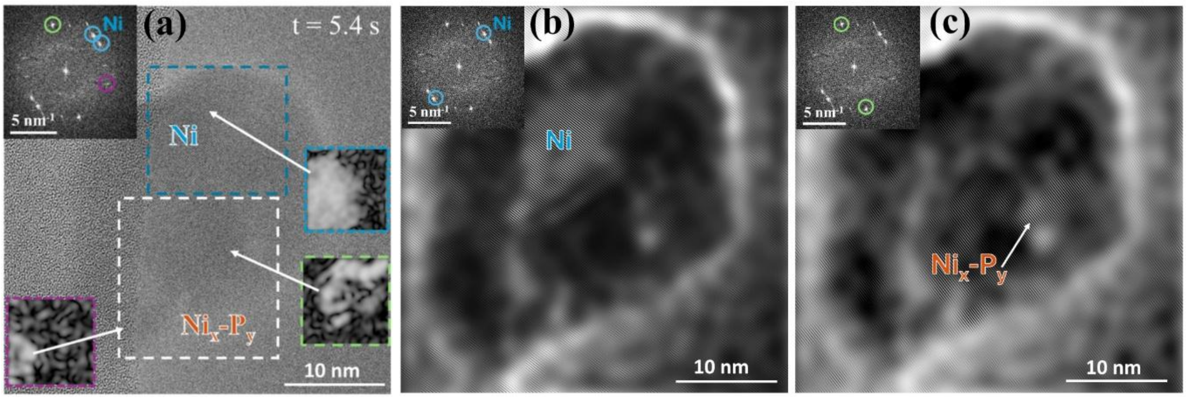

Figure S6. Inverse power spectra analysis of the regions highlighted in figure 2c. Spot masks were applied to isolate the initial Ni reflections (two distinct reflection sets from Ni are marked in blue color) and newly emerged reflections from nickel phosphide domains (marked in green color). The corresponding inverse power spectra analysis isolates the crystalline Ni containing region (S6 b) and the newly emerged Ni_xP_y domains (S6 c). This confirms the early growth of Ni_xP_y domains from the

nucleation sites. Movie conditions: T = 300 °C, PH_3 partial pressure = 1.31 Pa, electron dose rate ≈ 3.4 x 10^3 e^- $Å^{-2}$ s^{-1} (dwell time: 0.5 s).

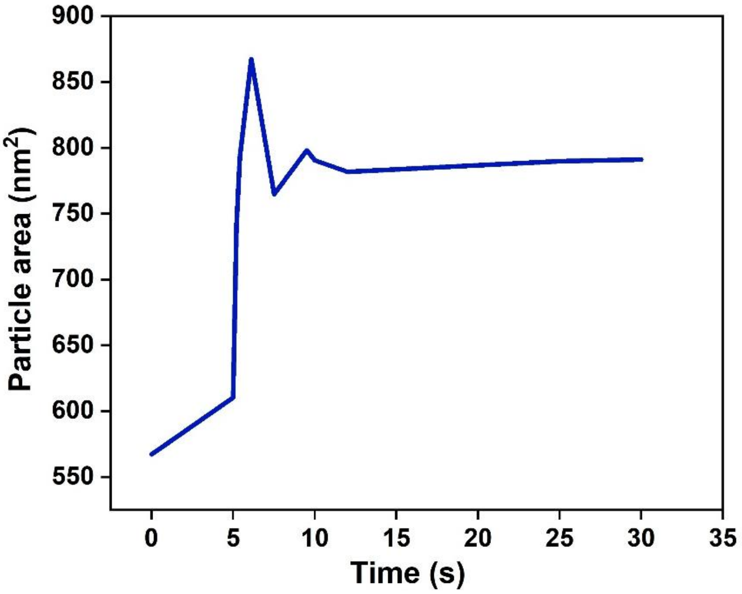

Figure S7: Projected particle area as a function of time extracted from the *in situ* ETEM movie (Movie S1), showing the evolution of the Ni nanoparticle under low PH_3 pressure at 300°C. The initial rapid increase in the projected area corresponds to the growth of Ni_xP_y domains under PH_3 environment, followed by a gradual stabilization associated with completion of the phase transformation and structural relaxation. The particle area was estimated using ImageJ by tracking the particle outline as a function of time.
